# Do Italian women prefer cesarean section? Results from a survey on mode of delivery preferences

**DOI:** 10.1186/1471-2393-13-78

**Published:** 2013-03-26

**Authors:** Maria Regina Torloni, Ana Pilar Betrán, Pilar Montilla, Elisa Scolaro, Armando Seuc, Agustina Mazzoni, Fernando Althabe, Francesca Merzagora, Gian Paolo Donzelli, Mario Merialdi

**Affiliations:** 1 Obstetrics Department, São Paulo Federal University, São Paulo, Brazil, Rua Borges Lagoa 564, conj. 63, São Paulo, SP, CEP 04038-000, Brazil; 2 Department of Reproductive Health and Research, World Health Organization, 20, via Appia CH-1211, Geneva, Switzerland; 3 O.N.Da, Osservatorio Nazionale sulla salute della Donna, Milan, Italy, Foro Buonaparte, Milan, 48 2012, Italy; 4 Institute for Clinical Effectiveness and Health Policy, Buenos Aires, Argentina, Dr. Emilio Ravignani 2024, Buenos Aires, C1414CPV, Argentina; 5 Meyer Children Hospital, University of Florence, Italy, Viale Gaetano Pieraccini, Florence, 24 50141, Italy

**Keywords:** Health surveys, Delivery, Obstetric, Cesarean section, Vaginal birth after cesarean, Public opinion, Women

## Abstract

**Background:**

About 20 million cesareans occur each year in the world and rates have steadily increased in almost all middle- and high-income countries over the last decades. Maternal request is often argued as one of the key forces driving this increase. Italy has the highest cesarean rate of Europe, yet there are no national surveys on the views of Italian women about their preferences on route of delivery. This study aimed to assess Italian women´s preference for mode of delivery, as well as reasons and factors associated with this preference, in a nationally representative sample of women.

**Methods:**

This cross sectional survey was conducted between December 2010-March 2011. An anonymous structured questionnaire asked participants what was their preferred mode of delivery and explored the reasons for this preference by assessing their agreement to a series of statements. Participants were also asked to what extent their preference was influenced by a series of possible sources. The 1^st^ phase of the study was carried out among readers of a popular Italian women´s magazine (*Io Donna*). In a 2^nd^ phase, the study was complemented by a structured telephone interview.

**Results:**

A total of 1000 Italian women participated in the survey and 80% declared they would prefer to deliver vaginally if they could opt. The preference for vaginal delivery was significantly higher among older (84.7%), more educated (87.6%), multiparous women (82.3%) and especially among those without any previous cesareans (94.2%). The main reasons for preferring a vaginal delivery were not wanting to be separated from the baby during the first hours of life, a shorter hospital stay and a faster postpartum recovery. The main reasons for preferring a cesarean were fear of pain, convenience to schedule the delivery and because it was perceived as being less traumatic for the baby. The source which most influenced the preference of these Italian women was their obstetrician, followed by friends or relatives.

**Conclusion:**

Four in five Italian women would prefer to deliver vaginally if they could opt. Factors associated with a higher preference for cesarean delivery were youth, nulliparity, lower education and a previous cesarean.

## Background

It is estimated that about 20 million cesarean section (CS) deliveries occur each year in the world [1,2] making this the most frequent abdominal surgery performed in adults. Despite the lack of scientific evidence indicating major benefits of delivering though CS for nonmedical reasons, and increased risks for mother and infants in this situation [3-6], the rates of CS have steadily increased in almost all middle- and high-income countries over the last three decades. According to the latest global estimates, the average CS rate is approximately 15%, with large discrepancies between and within different countries [1,7]. While several African countries have CS rates as low as 1-2% [1,8], between 20-30% of all deliveries in the United States and Canada are by CS, and in several Latin American countries CS rates exceed 40%, reaching 80% in the private sector [1,9,10]. Latest estimates indicate that in 2009, 39% of all women in Italy delivered by CS [11], making it the European country with the highest rate of CS.

In order to develop and implement safe and successful strategies for the reduction of unnecessary CS, it is essential to investigate the factors contributing to this trend, in particular in settings with high CS rates. Maternal request is often pointed as one of the key forces driving the worldwide CS increase [12-17]. Paradoxically, the existing evidence from surveys conducted to date indicates that the vast majority of women express a clear preference for vaginal delivery [18-20]. In Italy, maternal request accounted for only 6.4% of all the CS performed between 1996–2000 in one university hospital, although the rates increased significantly from 4.5% to 9.0% over the five year period [21]. To better understand the reasons for these controversies and to identify modifiable factors potentially involved in rising rates of CS, it is important to obtain the views of a representative sample of women. In Europe, most of the surveys on views about route of delivery have been conducted in Nordic countries or the UK [22-29], where CS rates are among the lowest in Europe. The only two publications from Italy [30,31] covered only a very specific group of women, i.e. healthy pregnant multiparas without a previous CS or primiparas in the immediate post-partum period, thus compromising the generalizability of their findings. In addition, they were conducted approximately 10 years ago. Since then, the national CS rate has largely increased, despite substantial regional variation, with rates ranging from 27.4% in the northern region to 45.4% in southern Italy [32]. The scarce literature on the delivery preferences of a representative sample of women in the country with the highest CS rate of Europe motivated us to perform this study.

The aim of this study was to assess what is the preferred mode of delivery, as well as the reasons and factors associated with this preference, in a nationally representative sample of Italian women.

## Methods

This study was developed in two phases. Initially, a survey was carried out among the readers of a popular Italian women´s magazine (*Io Donna*). An initial analysis of the characteristics and place of residence of the *Io Donna* respondents revealed that most were women from the region of Milan. Since there are large regional variations in CS rates in Italy and we wanted to obtain the views of a nationally representative sample of women, we went on to the 2^nd^ phase of the study and conducted phone interviews with women from all other areas of Italy excluding the Milan region.

### Survey through *Io Donna* magazine

*Io Donna* is an Italian magazine published as a weekly Saturday supplement with *Il Corriere de la Sera*, one of the oldest and most reputable Italian newspapers. With 422,000 weekly copies, *Io Donna* has an estimated 1,468,000 readers, about 80% of which are adult women [33]. On its December 18^th^ 2010 issue, this magazine published an article on the rising rates of CS in Italy, despite governmental efforts to curb this trend and recommendations of the World Health Organization. The article also mentioned that CS rates were lower in other European countries and described potential reasons for these differences, such as older maternal age, hospital policies and lack of options. The study´s one-page questionnaire was available alongside the article, to be completed online or by hand and mailed by interested readers (see Additional files 1 and 2). The call to participate in the survey was also posted on the *Io Donna* web site on the same date and the questionnaire remained available online for two and a half months, until March 1^st^ 2011. Women could therefore participate via regular postal mail or internet. Questionnaires were anonymous but collected information on socio demographic characteristics. All women returning the questionnaire were included in the analysis.

### Telephone interviews

The 2^nd^ phase of the survey (telephone interviews) was designed and undertaken by Cegedim Strategic Data, a marketing research institute based in Milan using a computer-assisted telephone interviewing (CATI) system. This is a telephone surveying technique in which the interviewer follows a script provided by a software application that manages call scheduling, geographical distribution, quota control, disposition monitoring and call organization. Respondents were selected through a geographically stratified random digit dialing approach. The sample size of 750 adult women age (20-40) years was based on geographic area and population size representativeness, excluding the Milan region which had already been represented in the magazine survey. Adult women fluent enough in Italian to hold the interview were considered eligible for inclusion. Italian nationality was not part of the inclusion requirements as health services coverage is universal in Italy and all women, regardless of nationality or legal status, have the right to receive health care and assistance without charge, if presenting to a health facility.

The telephone interviews were conducted during a one-week period, from March 1^st^ to 7^th^ 2011, in the afternoons and evenings, by 12 trained professional interviewers. Each interview lasted an average of 5 minutes. Before answering the questionnaire, participants were informed about its purpose and its anonymous and voluntary nature. No incentives were offered to participate.

The telephone interviewers entered the responses of the participants directly in an electronic spreadsheet. A data quality assurance process was undertaken and interviews with more than two blank fields were excluded.

### Survey instrument and analyses

The survey instrument used in both phases was the same and consisted of four sections (Additional files 1 and 2). The first section consisted of a closed question asking what was the participants preferred mode of delivery with two possible answers: vaginal or cesarean. The 2^nd^ section consisted of several statements used to explore the reasons for preferring each of the two routes, with independent statements for each route. The responses to each of these statements were measured using a 5-point Likert scale with 1 corresponding to “strongly disagree” and 5 to “strongly agree”. The third section consisted of a list of eight potential sources of influence on the participants´ preference, including persons (such as health professionals, family members and friends and courses or media (such as internet, television or magazines) which respondents were asked to tick, as appropriate. The fourth section of the questionnaire collected information on the participant´s sociodemographic characteristics (age and education) as well as her obstetric history (parity and previous routes of delivery).

The original questionnaire was developed by a team of investigators experienced in public health and surveys. For the layout of the questionnaire, the team worked with communication and design professionals from *Io Donna* to produce a clear, reader-friendly and attractive survey tool. The original questionnaire was tested on 10 female volunteers and modified to ensure that instructions were clear and unambiguous, that the meaning of the questions was the same for all respondents, that there were sufficient response categories available for the 2^nd^ section (reasons for preferring vaginal birth or CS) and that no question was systematically missed by the respondents. The final version was tested for face and content validity on 15 other women.

The data were analyzed descriptively and analyses were performed using SPSS software. Chi squared test was used to compare differences in categorical variables. P<0.05 was considered significant.

This study was a collaboration between the NGO ONDa (Osservatorio Nazionale sulla salute della Donna), the magazine *Io Donna* and the Department of Reproductive Health and Research at the World Health Organization to gain insight on route of delivery decision.

All respondents were provided with clear information about the nature of the data being collected, the purpose for which it was going to be used and the identity of the organization holding the data. Respondents could withdraw their consent at any time by refusing to cooperate in the phone interview. This anonymous opinion survey was conducted in accordance with the standards of ethical conduct proposed by the ICC/ESOMAR Code for social and market research [34]. The study was approved by the ethics´ committee of the University of Florence.

## Results

The survey included 1000 women, 250 of them recruited through the web page of the woman´s magazine *Io Donna* (246 via internet and 4 via postal mail) and 750 through telephone interviews. Most of the participants (53.1%) were between 25–35 years of age, had secondary education (58.6%) and had given birth to at least one baby (53.7%). Among the 537 who had previously given birth, 207 (38.5%) had experienced a CS and 87% of these women (180/207) responded that the decision to perform the CS had been made by their attending doctor, for medical reasons.

Overall, 80% of the participants declared that they would prefer a vaginal birth, if they could opt for route of delivery. Preference for CS was higher among younger women; almost 35% of those < 25 years preferred a CS compared to less than 16% of those > 35 years (Table 1). Similarly, the proportion of women with lower education who preferred CS was significantly higher than those with higher education (20.5% versus 13.4%). Women with a previous delivery were more likely to prefer vaginal delivery than those without any deliveries (82.3% versus 77.3%). Within the group of over 500 women with previous births, those without a previous CS were even more likely to prefer a vaginal delivery than women who had undergone at least one previous CS (94.2% versus 60.0%, respectively).

**Table 1:** Characteristics of 1000 Italian women according to preferred mode of birth

**Characteristics**	**Prefer VD N=799**	**Prefer CS N=201**	**All**	**P**
Age, years				
< 25	92 (65.2)	49 (34.8)	141	
25-35	430 (81.0)	101 (19.0)	531	
>35	276 (84.7)	50 (15.3)	326	<0.001
Education				
1^ary^	97 (79.5)	25 (20.5)	122	
2^ary^	447 (76.3)	139 (27.3)	586	
University	254 (87.6)	30 (13.4)	290	< 0.001
Ever given birth				
No	357 (77.3)	105 (22.7)	462	
Yes	441 (82.3)	95 (17.7)	536	< 0.001
Among women who have given birth:				
At least 1 previous CS^1^	131 (60.0)	76 (40.0)	207	
No previous CS	309 (94.2)	19 (5.8)	328	0.049

CS: Cesarean section. VD: Vaginal delivery. All values expressed as N (%).

^1 ^Percent calculated over 535 women with previous births who answered this question.

**Figure 1:**
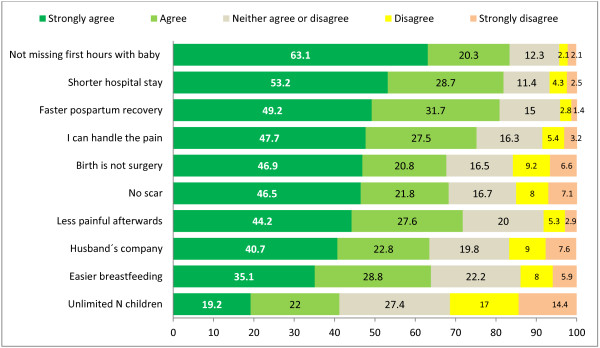
**Main reasons for preferring a vaginal delivery according to 800 Italian women. **N: Number.

Over 80% of the participants strongly agreed or agreed with the statement that they preferred a vaginal birth because they did not want to miss the first hours of life with the baby and because the hospital stay was shorter in this type of delivery (Figure 1). The 3^rd^ statement with the highest proportion of agreement (75.2%) for preferring a vaginal delivery was that the woman knew she could handle the pain involved. Other statements with high rates of agreement (approximately 70%) were that vaginal birth was a more natural, non-surgical, way of delivering, that it left no scar and that it was less painful in the post-partum period. A somewhat smaller number of participants (64%) preferred a vaginal delivery because the husband could be present or because it made breastfeeding easier. Less than half (41.2%) of the participants agreed that their preference for a vaginal birth was because it allowed them to have a larger number of babies than CS.

**Figure 2:**
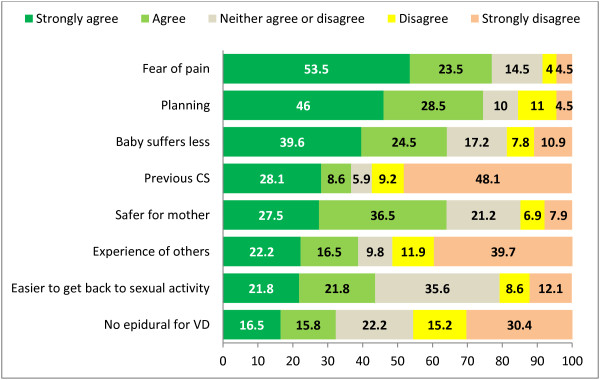
**Main reasons for preferring a cesarean delivery according to 200 Italian women. **CS: Cesarean section, VD: Vaginal delivery.

Among women preferring a CS, 77% strongly agreed or agreed that they feared the pain of birth and 74.5% strongly agreed or agreed that a reason for preferring CS was the convenience of scheduling the delivery (Figure 2). Almost two-thirds (64%) of the women who preferred a CS strongly agreed or agreed that this route of delivery was safer for the mother and that it caused less suffering for the baby. Approximately 40% agreed that they preferred a CS because it allowed a quicker return to sexual activity, or because they had good reports from friends who had delivered through this route or because they themselves had a previous CS. The statement with the lowest rate of agreement among women who preferred to deliver through CS was because the hospital offered no epidurals for vaginal deliveries (32.3%).

Statements with highest levels of disagreement for preferring CS were: having had a previous CS (57.3% of women strongly disagreed or disagreed), the experience reported by others (51.6%) and not having the epidural available for the vaginal delivery (45.6%).

**Figure 3:**
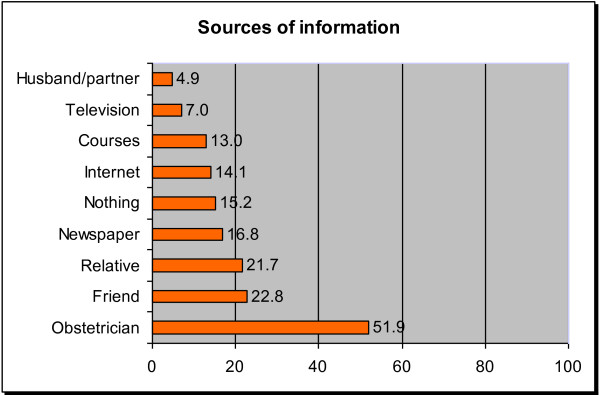
**Main sources of influence on the preferred mode of delivery of 1000 Italian women.** Values expressed as percentages.

Over half of the participants responded that their obstetricians were an important source that influenced their preferred route of delivery (Figure 3). Other important influences were friends or relatives, cited by approximately 22% of Italian women. Various forms of media or public resources were also reported to influence preferred route of delivery; newspapers were the most influential (17%) and television the least influential (7%). Only 5% of women indicated that their husbands or partners had influenced their preferences.

## Discussion

According to this survey, 4 in 5 Italian women would prefer to deliver vaginally if they could opt. The preference for vaginal birth was higher among older, more educated and multiparous women, and especially among those without any previous CS. The main reasons for preferring a vaginal delivery were not wanting to miss the first hours of life of the baby, a shorter hospital stay and faster postpartum recovery. The main reasons for preferring a CS were fear of pain, convenience to schedule the delivery, and because it was perceived as being less traumatic for the baby. The source which influenced the preference of more women was their obstetrician, followed by friends or relatives.

This survey obtained the views of 1000 Italian women recruited initially through a web survey that was then complemented by a phone survey, in order to obtain a final dataset that was representative of all the geographic regions of Italy. The answers obtained from both samples were combined for our analyses. This type of approach, mixing web and telephone surveys, is commonly used in market research and ensures response rates that are significantly higher than when using exclusively telephone or web survey [35].

Approximately half of the participants were multiparous, with almost 40% of them reporting at least one previous CS, in line with the most recent national CS rate estimates [11].

This survey complements and adds to the two previous Italian studies, bringing a representative and current picture about delivery preferences. The previous Italian studies were conducted many years ago and interviewed only pregnant or recently delivered women, which is an important selection bias. In 1999, Donati et al. [30] interviewed 1019 primiparas without any medical or obstetric complications on the eve of their discharge in 23 university hospitals located predominantly in southern regions of Italy. While the overall rate of CS among the participants was 36%; only 14.7% of these women declared that they would have opted for this route, a rate similar to the 17.7% preference for CS among our 536 women with at least one previous delivery. More specifically, those investigators report that 91% of the women who had just delivered vaginally and 73% of those who had a CS would have preferred a vaginal birth. Our findings were similar, with 94.2% of our participants without previous CS preferring a vaginal delivery, compared to 60.0% of those with at least one CS (Table 1). The very specific characteristics of the participants and the tertiary nature of the hospitals preclude generalization of the findings of that study to the rest of the Italian female population. Similar methodological limitations apply to the study by Mancuso et al. [31], which interviewed 390 healthy pregnant women without a previous CS, who were being managed by six obstetricians at a single hospital in Sicily between 2004–2005. Overall, 16.9% of those participants declared that they would prefer to have a CS, a rate substantially higher than the 5.8% among our 328 women without a previous CS. This is probably due to the fact that while our sample included women from all over the country, that study interviewed only women living in Southern Italy where CS rates are substantially higher than the national average. According to the authors, factors associated with CS preference included age >35 years, high level of education, previous infertility and smoking. Important differences in the study populations and methods make it difficult to compare our results with the findings of this publication.

Several factors contribute the high CS rates in Italy. Although 88% of all Italian birth centers are public and provide deliveries free of charge, approximately 60% of all CS occur in private centers, especially in those with less than 1000 deliveries per year. Similarly, a large proportion of the 40% of all CS performed in public facilities occur in the settings with less than 1000 deliveries per year [36]. Therefore, it seems that the high CS rate in Italy is due to the number of CSs performed in private centers and public centers with less than 1000 deliveries. The high rate of CSs may also be due to higher reimbursement rates for cesarean deliveries and to the lack of continuous availability of epidural anesthesia for vaginal delivery in all birth centers [36].

Our 20% CS preference rate was higher than the overall 16% rate reported by a recent systematic review on women´s preferences which analyzed 39 studies involving over 17,000 women across a range of countries, and almost twice the rate for European women in the same review, which was 11%, according to12 studies involving over 10,000 women [19]. Our findings of a substantially higher CS preference may in part be explained by the increasing perceived safety of CS in relation to vaginal delivery, especially in a country with a high CS rate. Indeed, in our study, approximately two thirds of the Italian women who preferred CS strongly agreed or agreed with the statement that CS is safer for the mother and less traumatic for the baby. Continued advances in surgical and anesthetic techniques, along with the availability of antibiotics and blood transfusions, have made cesareans a much safer intervention for the average woman of today. Although the relative risks for complications in an elective CS are still several times higher than in a vaginal delivery (3;37), the absolute risks for significant maternal or perinatal morbidity and mortality are very small and may contribute to the sense of safety of this surgery and to the rising rates of CS performed due to maternal request.

While 40% of our Italian women with a previous CS declared that they would prefer to have another CS, less than 6% of those with only vaginal deliveries would prefer a CS. The systematic review mentioned above reported similar results in a worldwide analysis: almost 30% of multiparas with a previous CS preferred this route, compared to 10% of those without that experience [19]. If, as some suggest, maternal request is one of the factors driving the increase in CS rates worldwide [21,38,39], these findings underscore the importance of avoiding the first CS, in order to decrease the overall CS rates in any population. Given that this survey did not investigate the reasons or the moment (antepartum or intrapartum) for the previous CS, we cannot infer about the possible traumatic experience that women currently preferring a CS may have had in their previous birth experience. A negative previous birth experience has been described as a predictor for preferring CS [40] and this is reinforced by the large proportion of women in our survey citing fear of pain as a major reason for preferring CS. Interestingly, a large proportion of our respondents (57%) who preferred a CS did not feel that a previous CS is a reason *per se* for preferring a subsequent CS, which could indicate a certain degree of access to information about options of vaginal birth after CS.

The most cited reasons for which Italian women would prefer a CS in our survey were fear of pain and convenience. Fear of pain is an important dimension of childbirth fear, along with other factors such fatigue, lack of support, daily stressors and maternal anxiety [41]. Fear of pain is a common reason for requesting a CS, especially among nulliparas and younger women [27,31,42-45]. Control issues and the possibility of scheduling the date of delivery through elective CS have also previously been reported as reasons for preferring this route [22,46,47].

Among a list of 10 reasons for preferring vaginal delivery proposed by the survey, the proportion of women who strongly agreed or agreed was high –between 63% and 80%– for all except one reason (see Figure 1). It is worth noticing that statement with the highest agreement rate did not refer to safety, obstetric or financial reasons, but to an emotional need of bonding with the baby as early as possible. The feeling of empowerment during labor and the sense of accomplishment afterward, despite having to endure pain, as well as the significance of an early and intense contact with the baby are being increasingly studied and documented [48-51]. Awareness and respect for emotional issues of women may play increasingly more important roles in delivery preferences in societies where obstetric safety and basic wellbeing during delivery are ensured. This is supported by our finding that, despite the fear of pain being cited as a major reason for wanting a CS, the lack of epidural for vaginal delivery was not seen as a reason for having a CS by the majority of the women (See Figure 2).

Obstetricians were by far the most important source of influence on women´s preferences, followed by friends and relatives. This underscores the important role of obstetricians in the process of a woman´s preference for mode of delivery. Doctors´ views on the mode of birth may be even more influential in women with a previous CS. In depths analyses from a qualitative study suggest that while most women wanted a vaginal delivery before having a CS, many felt that vaginal birth was unsafe and unachievable after their first CS and stated that their doctors recommended CS as the safest option for them, reinforcing their decisions [40]. It was surprising to see that husbands or partners were cited as the least influential source in Italian women´s preferences on route of delivery.

This is the first study to assess the preferences on mode of delivery of a representative sample of Italian women, the country with the highest CS rates in Europe and where latest estimates show no decrease in this tendency. A strong feature of this study is the use of a questionnaire that was carefully constructed, tailored and tested to the country´s local context. This study is an example of a collaboration between research, engaged civil society and media. Effective multidisciplinary partnerships and synchronized actions between all players are necessary to raise awareness in emerging issues, monitor trends and reach a large number of people with the correct message, in diverse media [52]. These coordinated efforts are particularly important in issues like CS where multifaceted strategies are required to challenge simultaneously several fronts and factors.

Although the telephone survey was carried out using a strict sampling procedure, respondents could refuse to answer the questionnaire and it is possible that the characteristics of non-responders may differ from those of the participants. We also acknowledge the potential for self-selection and non-response bias in the magazine survey. The results of this study should be taken with caution. Although preferences of women are increasingly taken into consideration by obstetricians and have been pointed as a factor for increasing CS rates in some settings [53-55], it is unlikely that this is the only explanation for differences in CS rates between countries. The influence of Italian women´s preferences on the actual rates of CS in that country cannot be inferred from the data presented in this study. Future qualitative and quantitative studies are needed to address this association in Italy.

## Conclusion

One in every five Italian women expressed a preference for CS. Factors associated with this preference were younger age, nulliparity, lower instruction and a previous delivery through CS.

## Competing interests

The authors declare that they have no competing interests.

## Authors’ contributions

MRT performed the analyses and drafted the manuscript. APB conceived the study, participated in the design of the study, performed analyses and drafted the manuscript. PM participated in the design of the study, organized and supervised data collection. ES participated in data collection, management and analyses. AS participated in the design of the study and data management and performed statistical analyses. AM and FA helped to develop and test the questionnaire used and participated in data analyses. FM participated in data collection and organization. MM participated in the conception and design of the study and coordinated data management and analyses. All authors read and approved the final manuscript.

## Pre-publication history

The pre-publication history for this paper can be accessed here:

http://www.biomedcentral.com/1471-2393/13/78/prepub

## Supplementary Material

Additional file 1 **Questionnaire on Italian women´s preferences for route of delivery. **Original online questionnaire provided by *Io Donna *magazine to get readers´ opinion. Click here for file

Additional file 2 English version of questionnaire on Italian women´s preferences for route of delivery. Click here for file
