# Supplementary material for: Do Italian women prefer cesarean section? Results from a survey on mode of delivery preferences
Source: BMC Pregnancy Childbirth. 2013 Mar 26;13:78. doi: 10.1186/1471-2393-13-78 (PMC3621281; doi:10.1186/1471-2393-13-78)
Supplement: Additional file 1 — Questionnaire on Italian women´s preferences for route of delivery. Original online questionnaire provided by Io Donna magazine to get readers´ opinion. [file 1471-2393-13-78-S1.pdf]

Naturale o cesareo, il parto che divide

L'Italia ha il primato di nascite con intervento chirurgico. Diteci cosa ne pensate: ecco il sondaggio di *Io donna* e *Onda*

di Elena Meli

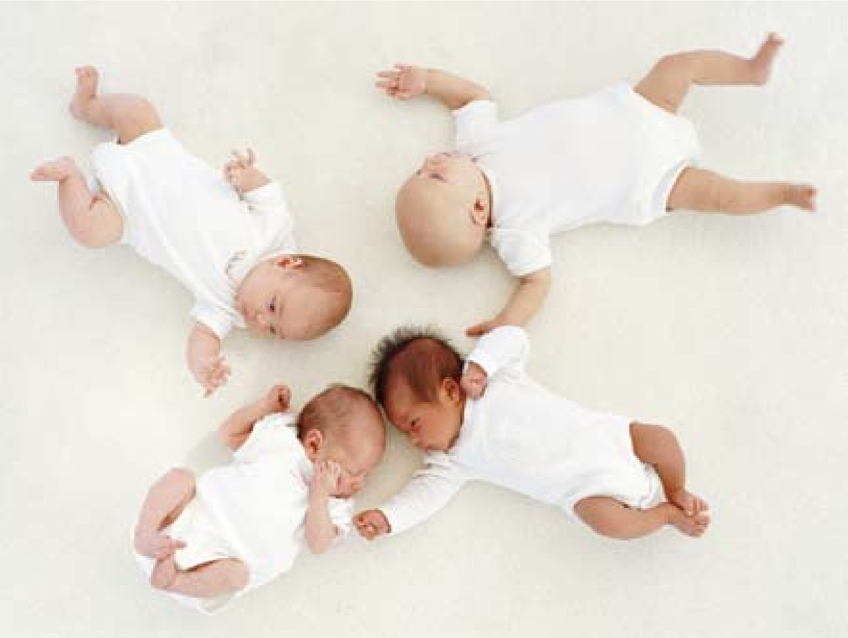

Corbis

Lo conferma l'Agenzia nazionale per i servizi sanitari regionali (*agenas.it*): il primo vagito arriva in sala operatoria invece che in sala parto per quattro bambini italiani su dieci. E il numero di cesarei, a dispetto delle politiche di contenimento più annunciate che realizzate, ha continuato a salire in quasi tutta Italia anche nel 2009.

«Per questo abbiamo deciso di dare voce alle donne, lanciando insieme con *Io donna* un grande sondaggio che le vede protagoniste in prima persona: vogliamo capire dalle lettrici, che sono già diventate madri o che pensano di diventarlo in futuro, quali sono i reali motivi che le hanno o che le potrebbero spingere a preferire al parto naturale quello tramite taglio cesareo. I dati che raccoglieremo sono essenziali per poter studiare la strategia migliore che faccia invertire questa tendenza, uno degli obiettivi della collaborazione in atto con l'Organizzazione mondiale della Sanità» dice Francesca Merzagora, presidente di Onda, l'Osservatorio sulla salute della donna (*ondaosservatorio.it*).

In Campania il numero di cesarei sfiora il **60 per cento**, in Sicilia supera il 50 per cento e in tutte le Regioni del Sud oltrepassa il 40 per cento; soltanto Friuli Venezia Giulia e Trentino Alto Adige si avvicinano alla soglia del 20 per cento raccomandata dal ministero della Salute.

In alcune cliniche private e ospedali con meno di 500 parti all'anno, si registrano punte che vanno oltre l'80 per cento: cifre allarmanti, perché un cesareo, praticato senza necessità mediche, duplica il rischio di mortalità per madre e bambino. «Secondo l'Oms, i cesarei non dovrebbero essere **più del 15 per cento** dei parti totali» spiega Pilar Montilla, responsabile per la ricerca e i progetti internazionali di Onda. «Alcuni Paesi europei, come Olanda (14 per cento) e Francia (18 per cento), rispettano la soglia dell'Oms; oltrepassarla di poco può essere il segnale di maternità più tardive rispetto al passato. Ma quando i numeri sono quelli dell'Italia, che detiene in assoluto il primato europeo di cesarei, è inconfutabile indizio di una patologia del sistema. Al Sud spesso è colpa della scarsa organizzazione delle strutture, che non garantiscono l'epidurale 24 ore su 24, e dell'inadeguato monitoraggio da parte delle autorità sanitarie. Molti cesarei, inoltre, sono dovuti a un eccesso di cautela dei medici, soprattutto quando l'ospedale non è in grado di assicurare un parto naturale in totale sicurezza». Ma anche al Nord, le donne spesso chiedono il cesareo perché è più comodo, semplice e indolore. Oppure anche perché nessuno propone loro un'alternativa.

«Il cesareo è pur sempre un intervento chirurgico, che **comporta una degenza più lunga**, dolore postoperatorio, il rischio di doverlo ripetere anche per i parti successivi: promuovere il parto naturale è indispensabile, sia per la madre, sia per il bambino» conclude Merzagora. ●

RISPONDETE SU IODONNA.IT MA ANCHE PER LETTERA, FAX O EMAIL

Per sapere che cosa sanno e che cosa pensano davvero le italiane del parto cesareo, *Io donna* e Onda, l'Osservatorio sulla salute della donna, lanciano un grande sondaggio tra le lettrici. Che sono tutte invitate a offrire il loro contributo compilando questo questionario, completamente anonimo (i dati demografici servono solo a fini statistici). Unica richiesta: la massima immediatezza e sincerità nelle risposte.
Il questionario può essere compilato on line su:
- leiweb.it/iodonna/sondaggio-parto.shtml
oppure inviato per posta a:
- Onda, Osservatorio nazionale sulla salute della donna, Foro Bonaparte 48, 20121 Milano
oppure per fax, sempre a Onda:
- al numero 02.29004729
oppure via mail a:
- sondaggi@ondaosservatorio.it

La valutazione va da 1 (in totale disaccordo) a 5 (in totale d'accordo)

1) Se dovessi partorire, come sceglieresti di farlo?

- ☐ Parto naturale
- ☐ Parto cesareo

2) Perché sceglieresti il taglio cesareo?

- Ho troppa paura del dolore 1 2 3 4 5
- Posso programmare il parto 1 2 3 4 5
- Mio figlio soffrirà di meno 1 2 3 4 5
- L'ospedale non dispone dell'epidurale 1 2 3 4 5
- Ho già partorito con un cesareo 1 2 3 4 5
- È più facile riprendere la vita di coppia 1 2 3 4 5
- L'ha già fatto una mia amica 1 2 3 4 5
- È più sicuro per la mia salute 1 2 3 4 5

3) Perché sceglieresti il parto naturale?

- Non rimane nessuna cicatrice 1 2 3 4 5
- Non voglio perdere le prime ore di vita di mio figlio 1 2 3 4 5
- Il post operatorio è meno doloroso 1 2 3 4 5
- Ho paura del dolore, ma ce la posso fare 1 2 3 4 5
- Non voglio che mio figlio nasca con un intervento chirurgico 1 2 3 4 5
- Non avrò difficoltà ad allattare 1 2 3 4 5
- Così mio marito può assistere al parto 1 2 3 4 5
- Voglio avere molti figli e so che, con il cesareo, il numero è limitato 1 2 3 4 5
- Il recupero post parto è più rapido 1 2 3 4 5
- La degenza in ospedale è più breve 1 2 3 4 5

4) Chi ti ha fornito informazioni sul tipo di parto in modo da aiutarti nella scelta?

- ☐ Il mio medico o l'ostetrica
- ☐ Mio marito / il mio partner
- ☐ Un parente
- ☐ Una mia amica
- ☐ Internet
- ☐ Televisione
- ☐ Corsi
- ☐ Riviste

INFORMAZIONI SOCIO-DEMOGRAFICHE:

Età:
- ☐ < 25 anni
- ☐ 25-35 anni
- ☐ > 35 anni

Istruzione:
- ☐ Primaria
- ☐ Secondaria
- ☐ Universitaria

Hai mai partorito?:
- ☐ Sì
- ☐ No

Hai mai avuto un taglio cesareo? Se sì, per quale motivo?:
- ☐ L'ho voluto io
- ☐ L'ha deciso il medico per ragioni cliniche

Il sondaggio è su iodonna.it
