# Supplementary material for: Do Italian women prefer cesarean section? Results from a survey on mode of delivery preferences
Source: BMC Pregnancy Childbirth. 2013 Mar 26;13:78. doi: 10.1186/1471-2393-13-78 (PMC3621281; doi:10.1186/1471-2393-13-78)
Supplement: Additional file 2 — English version of questionnaire on Italian women´s preferences for route of delivery. [file 1471-2393-13-78-S2.pdf]

Vaginal or cesarean delivery preferences (English translation)

Answer the first question then select the number (from 1 to 5), that best describes how you feel. Number 1 means that you strongly disagree with the statement, Number 5 means that you strongly agree with the statement.

1. If you were to have a baby, how would you prefer to deliver?

□ By vaginal delivery

□ By caesarean section

2. Why would you prefer caesarean section?

- Because I am afraid of pain 1 2 3 4 5
- With a caesarean section, I can plan when to deliver. 1 2 3 4 5
- With a caesarean section, my baby suffers less. 1 2 3 4 5
- The hospital does not offer epidurals for vaginal delivery. 1 2 3 4 5
- I have already had a caesarean section. 1 2 3 4 5
- After a caesarean it is easier to go back to a couple´s life. 1 2 3 4 5
- A friend of mine has already had a caesarean section. 1 2 3 4 5
- A caesarean section is safer for my health. 1 2 3 4 5

3. Why would you prefer vaginal delivery?

- Becasuse it does not leave any scar. 1 2 3 4 5
- I don't want to miss the first hour of life of my baby. 1 2 3 4 5
- The postpartum period is less painful. 1 2 3 4 5
- I am afraid of pain but if other women have gone through it, I can also handle it. 1 2 3 4 5
- I don't want my baby to be born through a surgical procedure. 1 2 3 4 5
- It will be easier for me to breastfeed. 1 2 3 4 5
- My husband/partner can be present at the delivery. 1 2 3 4 5

- I want to have many children and I know that with caesareans, the number is limited. 1 2 3 4 5

- The post-partum recovery is faster. 1 2 3 4 5

- The length of hospital stay is shorter. 1 2 3 4 5

4. How did you obtain the information about delivery that helped you to decide on your preference? (tick the boxes that apply)

• My obstetrician or midwife	◻	The internet	◻
• My husband/partner	◻	Television	◻
• A parent	◻	Through a course	◻
• A friend	◻	Newspapers/magazines	◻

Socio-economic characteristics:

- Age :
- Education:
- Have you ever had a baby?
- Have you ever had a caesarean delivery?
